# Diagnostic errors in Dentistry, opinions of egyptian dental teaching staff, a cross-sectional study

**DOI:** 10.1186/s12903-022-02565-9

**Published:** 2022-12-20

**Authors:** Naglaa El-Wakeel, Naglaa Ezzeldin

**Affiliations:** 1 Oral medicine and Periodontology department, Faculty of Dentistry, Al-Azhar University (Girls Branch), Cairo, Egypt; 2 Pediatric Dentistry, Faculty of Dentistry, October University for Modern Sciences and Arts, Cairo, Egypt

**Keywords:** Diagnostic errors, Dentistry, Teaching staff, Patient safety.

## Abstract

**Background:**

Diagnostic errors is a known problem in healthcare practice. Data on diagnostic errors in the dental field are extremely lacking. The objective of the study is to explore the perception of dental teaching staff about the prevalence of dental diagnostic errors in Egypt, identify the most commonly misdiagnosed dental conditions and point out the contributing factors and levels of patient harm.

**Methods:**

A cross-sectional questionnaire-based study was conducted on 151 dental teaching staff of Egyptian governmental and private universities. The questionnaire was distributed electronically via social media and messaging apps to dental staff members with at least five years of clinical experience to assess their opinion regarding the study objectives. Results were collected and statistically analyzed.

**Results:**

94.7% of participants believed that diagnostic errors represent an urgent problem, lecturers believed by 2.703 folds more than professors that diagnostic errors are an urgent problem The percentage of diagnostic errors was estimated to be < 20% and 20–40% by more than 90% of participants. The most commonly misdiagnosed conditions were oral mucosal lesions (83.4%), followed by temporomandibular joint and periodontal conditions (58.9%) for each. More than half of the participants (60.9%) believe that medical education methodology is one of the factors that lead to dental diagnosis errors. For the impact of errors on patients, 53% of participants reported moderate impacts followed by minor impact (37.7%) while 4.6% reported no impact and the same percentage reported major impact.

**Conclusion:**

This study with statistically significant results reported that dental diagnostic errors are frequent and need to be approached. Oral mucosal lesions, periodontal and temporomandibular joint diseases represent areas that include the most commonly seen errors. Further, besides the lack of resources, the dental education system and lack of proper training are the main causes of this problem.

**Supplementary Information:**

The online version contains supplementary material available at 10.1186/s12903-022-02565-9.

## Background:

Diagnostic errors causing patients harm from wrong or delayed testing or treatment have emerged as a global safety-recognized issue. Due to preventable morbidity and mortality, healthcare costs, and malpractice claims, The World Health Organization (WHO) pointed out the importance of diagnostic errors and prioritized safety areas in primary care [1, 2]. Diagnostic error is defined as: “failure to establish an accurate and timely explanation of the patient’s health problem(s) or communicate that explanation to the patient**”** [3]. This definition contains any failure in time access to care; recognizing the explanation of signs, symptoms, or diagnostic test results; listing possible differential diagnosis; in time follow-up and specialty referral [3, 4]. Compared with the many different safety concerns encountered in healthcare practice, diagnostic errors are perceived to be more likely to cause serious harm compared to other safety concerns [5, 6].

Despite numerous efforts and initiatives aiming to promote patients’ safety by reducing the occurrence of diagnostic errors, in dentistry, many patients suffer harm in dental clinics [7, 8]. Previous reports showed that there is an increase in dental complaints and negligence claims and 9% of these claims are related to diagnostic errors [9].

Although diagnostic errors may occur when symptoms and signs of disease are atypical or absent, they are largely related to cognitive-related factors (e.g., faulty data gathering or clinical reasoning, lack of knowledge or clinical experience) and systems-related factors (e.g., issues with policies, processes, and procedures, malfunctioning equipment) [10–13].

Different study approaches have been utilized to identify the breakdowns in the diagnostic process, the incidence of diagnostic errors, as well as contributing causes. These approaches include case reviews, surveying patients, voluntary reporting systems using standardized patients, diagnostic testing audits, and legal claims review [6, 14–16]. Not all these methods are feasible to implement, given limited time and resources [17]. Surveying clinicians/ dentists is one of the feasible approaches used to study various aspects related to diagnostic errors [3, 6, 18].

Extremely limited studies addressed dental diagnostic errors and how to avoid them. To the best of our knowledge, only one study addressed dental diagnostic errors from a broad perspective adopting dental teaching staff as a data source, using a convenience sample of 10 staff members [7]. Other studies investigated errors related to certain specified conditions like caries and traumatic injuries and only in specified dental specialties like pedodontics, oral surgery, and dental implants [6, 15, 16]. Thus, Information that holds reliable reflections related to the real situation concerning diagnostic errors in dental practice either locally in Egypt or worldwide is lacking. We surveyed clinicians with at least five years of clinical experience among dental teaching staff to explore their perceptions regarding various aspects related to dental diagnostic errors. Thus, our study aims to: 1- determine the frequency and prevalence of dental diagnostic errors, 2- identify the most missed diagnosed dental conditions, and 3- recognize contributing factors (highlighting medical education) and assess resulting patient harm.

## Methods:

### Participation and study design:

This online cross-sectional questionnaire-based study was undertaken in March and April 2022 using a sample of Egyptian dental teaching staff from governmental and private universities. Included participants were only dental teaching staff of clinical departments who have at least five years of experience. All procedures were conducted in compliance with the Helsinki Declaration and the research protocol was approved by the research ethics committee of the faculty of dentistry, Al-Azhar University - Girl’s branch, protocol code: REC-PD-22-06.

## Sample size calculation:

After reaching the final form of the questionnaire and for power analysis, a pilot study was carried out on 30 dentists to identify the percentage of believing that diagnostic errors represent an urgent problem. The percentage was 89.2%, using an alpha (α) level of (5%), an acceptable margin of error = 5%; the minimum estimated sample size was 148 participants. The data from the pilot study was not included in the final analysis. Sample size calculation was performed using Epi Info ^TM^ 7.2.2.2 for Windows.

## Questionnaire design and data collection:

The questionnaire was designed by the authors to cover the study objectives, guided by previously published relevant studies [3, 7]. Afterward, an independent committee including experts in community medicine, oral medicine, biostatistics, oral public health, epidemiology, and healthcare quality reviewed the questionnaire draft to assess the face and content validity. Experts did make some modifications as the biased, vague, and double questions were omitted. After a consensus was reached, the questionnaire form was finalized (Supplemental file 1).

The electronic questionnaire was conducted in English using Google form (www.googleforms.com). The questionnaire was distributed via social networks and messaging apps, A link was sent to teaching staff participants who fulfilled the inclusion criteria, and they were asked to disseminate the link to their colleagues with the same inclusion criteria. Multiple reminders were sent to encourage participation and unlimited time to complete was given, the estimated completion time was 7 min based on pilot testing. Before starting to fill out the questionnaire, participants were asked to review an introductory paragraph that included a definition of diagnostic errors [3], objectives of the survey, inclusion criteria, voluntary nature of participation, and ensuring the anonymity of respondents. Participant’s informed consent was obtained before answering the questionnaire by agreeing to participate. Only the authors had access to the collected data. The questionnaire consisted of three Sect. (15 questions):

Section 1: included items that explored the demographic and professional background of the participants, including academic degree, age, sex, dental specialty, and the average number of patients seen per week.

Section 2: included four closed-ended questions to assess if dental diagnostic errors represent an urgent problem to be addressed, the percentage of diagnostic errors in dental practice, and the areas in the dental field that include the most common diagnostic errors and their order according to the frequency of occurrence (top commonly seen, 2nd commonly seen, and 3rd commonly seen).

Section 3: included six questions to explore the opinion of participants regarding the top three possible causes of dental diagnosis errors and if they believe that medical education methods could play a role in the occurrence of diagnostic errors and who made the diagnostic errors (the participant himself or others). Moreover, the participant’s opinion regarding the impact of the error was explored by rating patient harm as minor (patient inconvenience, dissatisfaction, or increased length of dental procedures), moderate (short-term morbidity, need for either a higher level of care, or more invasive procedure), major (death, or permanent disability) [19].

The last question was an open-ended question to know if there are other problems related to dental clinical practice that need to be addressed by the decision-makers. After completing the required sample size, all responses were collected electronically via Google Form and were subjected to statistical analysis. Google forms was used as it provides accurate data collection, recording, and reporting as it decreases detection and reporting bias.

### Statistical analysis:

Qualitative data were presented as frequencies and percentages. The Chi-square test or Fisher’s Exact test was used to compare different degrees and different institutions. Binary logistic regression analysis was used to determine significant predictors of believing that diagnostic errors represent an urgent problem. The regression coefficient, standard error (SE), and 95% confidence interval (95% CI) were calculated. The significance level was set at P ≤ 0.05. Statistical analysis was performed with IBM SPSS Statistics for Windows, Version 23.0. Armonk, NY: IBM Corp.

## Results

### A. descriptive statistics

Demographic data:

The present study was conducted on 151 dental teaching staff: 54 males (35.8%) and 97 females (64.2%). Above 90% of the participants were aged 25–35 and 36–50 years old and only 7.3% were aged above 50 years old. All specialties were presented, and the most common specialty was oral medicine and periodontology. About half of the participants were from private universities and the other half were from governmental and Al-Azhar universities. More than half of the participants see an average of more than 30 patients per week **(**Table 1).

**Table 1:** Frequencies (n) and percentages (%) for demographic data of the study participants (n = 151)

Demographic data	n	%
Academic degree		
Professor	17	11.3
Associate professor	15	9.9
Lecturer	51	33.8
Assistant lecturer	68	45
Sex		
Male	54	35.8
Female	97	64.2
Age		
25–35 y	71	47
36–50 y	69	45.7
Above 50 y	11	7.3
Dental specialty		
Conservative dentistry	16	10.6
Dental biomaterials	2	1.4
Endodontic	5	3.3
Fixed prosthodontics	11	7.3
Oral biology	2	1.3
Oral medicine and periodontology	65	43
Oral radiology	4	2.6
Oral surgery	4	2.6
Orthodontics	5	3.3
Pathology	1	0.7
Pediatric Dentistry	26	17.2
Removable prosthodontics	10	6.6
Institution		
Governmental university	34	22.5
Private university	77	51
Al-Azhar university	40	26.5
Average number of patients / weeks		
10–20 patients	37	24.5
20–30 patients	26	17.2
More than 30 patients	88	58.3

Diagnostic errors:

The majority of participants believed that diagnostic errors represent an urgent problem (94.7%). The percentage of diagnostic errors was estimated to be < 20% and 20–40% by more than 90% of participants. The most diagnostic errors conditions were oral mucosal lesions (83.4%) while the least common was other conditions such as impacted teeth rather than third molar and referred pain. Oral mucosal lesions were also the top-seen conditions; followed by hard tooth structure related conditions while TMJ related conditions comprised the third most common condition (Table 2).

**Table 2:** Frequencies (n) and percentages (%) for answers to questions regarding diagnostic errors (n = 151)

Diagnostic errors	n	%
Do you believe that dental diagnostic errors represent an urgent problem that needs to be addressed?		
Yes	143	94.7
No	8	5.3
What is your estimate percentage of diagnostic errors you see in your clinical practice?		
< 20%	71	47
20–40%	67	44.4
40–60%	12	7.9
More than 60%	1	0.7
Which of the following areas/ aspects that include (comprise) the most dental diagnostic errors condition?		
Oral mucosal lesions Hard tooth structure related conditions TMJ related conditions Periodontal conditions Pulp therapy related conditions Other	126	83.4
	62	41.1
	89	58.9
	89	58.9
	79	52.3
	6	4
Among the areas you choose, please specify the most seen condition:		
Top commonly seen: Oral mucosal lesions	45	29.8
s commonly seen: Hard tooth structure related conditions	37	24.5
Third commonly seen: TMJ related conditions	46	30.5

Possible causes of diagnostic errors:

More than half of the participants (60.9%) believe that medical education methodology is one of the factors that lead to diagnostic errors in dentistry. The most common cause of diagnostic errors was misconduct in medical education methods; the second most common cause was lack of post-graduate training while the third common cause was lack of resources. One hundred and nine participants reported that they are responsible for the error and almost all of them reported their percentage of responsibility is a maximum of 40%. Regarding the responsibility of others, 136 participants reported other responsible parties for the error. Almost half of them declared that responsibility is 20–40%. Most participants reported minor and moderate impacts of the error, 4.6% reported no impact and the same percentage reported major impact (Table 3).

**Table 3:** Frequencies (n) and percentages (%) for responses to medical education questions (n = 151)

Medical education	n	%
Do you believe that medical education methodology is one of the factors that leads to diagnostic errors in dentistry?		
Yes	92	60.9
No	22	14.6
Don’t know	37	24.5
What are the possible causes of the diagnostic errors that you see in your clinical practice?		
Lack of resources required for proper diagnosis Lack of post-graduate training Misconduct in medical education methods for the undergraduate students Other	56	37.1
	82	54.3
	60	39.7
	6	4
Among the possible causes you chose, please specify the most common causes:		
Top cause: Misconduct in medical education methods	58	38.4
s commonly seen: Lack of post-graduate training	74	49
Third commonly seen: Lack of resources	75	49.7
Who is responsible for the error, please specify the percentage:		
Myself (n = 109)		
< 20%	88	80.7
20–40%	20	18.3
40–60%	1	0.9
> 60%	0	0
Other (n = 136)		
< 20%	35	25.7
20–40%	63	46.3
40–60%	24	17.6
> 60%	14	10.3
How serious was the clinical impact of the diagnostic errors? No impact Minor (patient inconvenience, dissatisfaction) Moderate (short-term morbidity, higher level of care, invasive procedure Major (death, permanent disability, or near life-threatening event)		
	7	4.6
	57	37.7
	80	53
	7	4.6

### B. Binary logistic regression analysis

A binary logistic regression model was constructed using believing that diagnostic errors represent an urgent problem (Yes/No) as the dependent variable and demographic data were the independent variables. Results of the regression model showed that degree and institution were statistically significant predictors for believing that diagnostic errors are an urgent problem. The results showed that lecturers believe 2.703 folds more than professors that diagnostic errors are an urgent problem (Odds Ratio = 2.703, P-value = 0.043) while participants of the private university are 0.113 less believing than those of Al-Azhar university that diagnostic errors are an urgent problem (Odds Ratio = 0.113, P-value = 0.040) (Table 4).

**Table 4:** Results of binary logistic regression analysis model showing predictors of believing that diagnostic errors is an urgent problem

Variables	Regression coefficient (b)	Standard Error (SE)	*P*-value	Odds Ratio (OR)	95% CI for OR
Sex	0.955	0.915	0.297	2.598	0.432–15.618
Age (Reference category: Above 50 y)					
25–35 y	-20.133	5.873	0.999	0.001	0.00001–3.212
36–50 y	-21.095	5.872	0.998	0.001	0.00001–3.21
Degree (Reference category: Professor)					
Assistant lecturer	2.282	2.008	0.256	3.798	0.191–10.395
Lecturer	3.122	1.543	0.043*	2.703	1.104–46.907
Associate professor	1.243	1.379	0.367	3.466	0.232–51.715
Institution (Reference category: Al-Azhar University)					
Governmental university	-0.872	1.166	0.455	0.418	0.043–4.112
Private university	-2.184	1.062	0.040*	0.113	0.014–0.903
Number of patients (Reference category: More than 30 patients)					
10–20 patients	18.66	4.915	0.998	0.001	0.00001–4.022
20–30 patients	1.327	1.224	0.278	3.77	0.342–4.552

*: Significant at P ≤ 0.05

Comparison between different degrees: A non-significant difference between answers to questions regarding diagnostic errors among different degrees was reported (Table 5).

**Table 5:** Results of comparison between answers to questions regarding diagnostic errors among different degrees

Diagnostic errors	Assistant Lecturer		Lecturer		Associate Professor		Professor		*P*-value
	n	%	n	%	n	%	n	%	
Do you believe that dental diagnostic errors represent an urgent problem that needs to be addressed?									
Yes	66	97.1	49	96.1	13	86.7	15	88.2	0.162
No	2	2.9	2	3.9	2	13.3	2	11.8	
What is your estimate percentage of diagnostic errors you see in your clinical practice?									
< 20%	30	44.1	25	49	6	40	10	58.8	0.574
20–40%	33	48.5	21	41.2	8	53.3	5	29.4	
40–60%	5	7.4	5	9.8	1	6.7	1	5.9	
More than 60%	0	0	0	0	0	0	1	5.9	
Which of the following areas/ aspects that include (comprise) the most dental diagnostic errors condition?									
Oral mucosal lesions Hard tooth structure related conditions TMJ related conditions Periodontal conditions Pulp therapy related conditions	15	22.1	18	35.3	5	33.3	7	41.2	0.792
	17	25	9	17.6	2	13.3	2	11.8	
	6	8.8	6	11.8	3	20	2	11.8	
	9	13.2	6	11.8	1	6.7	3	17.6	
	21	30.9	12	23.5	4	26.7	3	17.6	

*: Significant at P ≤ 0.05

Comparison between different institutions: There was a non-significant difference between answers to questions regarding diagnostic errors among different institutions except for the most diagnostic errors condition (P-value < 0.001) where at Al-Azhar and Governmental universities, oral mucosal lesions were the most diagnostic errors conditions followed by pulp therapy related conditions. At private universities, pulp therapy related conditions were the most diagnostic errors conditions followed by hard tooth structure related conditions (Table 6).

**Table 6:** Results of comparison between answers to questions regarding diagnostic errors among different institutions

Diagnostic errors	Al-Azhar University		Governmental Universities		Private Universities		*P*-value
	n	%	n	%	n	%	
Do you believe that dental diagnostic errors represent an urgent problem that needs to be addressed?							
Yes	38	95	30	88.2	75	97.4	0.127
No	2	5	4	11.8	2	2.6	
What is your estimate percentage of diagnostic errors you see in your clinical practice?							
< 20%	24	60	16	47.1	31	40.3	0.336
20–40%	15	37.5	14	41.2	38	49.4	
40–60%	1	2.5	4	11.8	7	9.1	
More than 60%	0	0	0	0	1	1.3	
Which of the following areas/ aspects that include (comprise) the most dental diagnostic errors condition?							
Oral mucosal lesions Hard tooth structure related conditions TMJ related conditions Periodontal conditions Pulp therapy related conditions	13	32.5	21	61.8	11	14.3	< 0.001*^†^
	4	10	3	8.8	23	29.9	
	7	17.5	3	8.8	7	9.1	
	5	12.5	3	8.8	11	14.3	
	11	27.5	4	11.8	25	32.5	

*: Significant at P ≤ 0.05, ^*†*^: *Chi-square test*

## Discussion:

Diagnostic errors in dentistry represent a hugely understudied issue in patient safety and more information is needed to augment efforts aiming to promote dental care quality [20]. The present study reports for the first-time frequency of diagnostic errors and areas in the dental field that incorporate the most frequent diagnostic errors. Further, this study highlighted some causes of this problem from the perspectives of dental teaching staff with a special reference to medical education. Although we cannot consider our sample to be perfectly representative of the universal pattern of diagnostic errors arising in dental clinical practice, it does offer what we consider- a close look into the types of errors, dentists are committing and observing. The 2020 diagnostic safety review recommends that healthcare organizations should begin to monitor diagnostic safety using the most robust data sources currently available, we considered teaching staff as the most reliable detectors of diagnostic errors due to experience gained from both teaching dental students and clinical practice [17].

Further, feedback from clinicians regarding diagnostic errors has been shown to offer a distinctive opportunity to explore both system-related and cognitive issues contributing to diagnostic errors [10, 21]. A minimum 5 years of clinical experience was required for participants because experience is vital to the process of decision-making [22], and when personally experiencing different diagnoses, this increases understanding of the conditions being identified and promotes the clinician’s ability to correctly diagnose them in the future [20, 22]. Another cause for including experienced teaching staff is that being an educator, they will be able to give their perceptions regarding the role of medical education methods in causing this problem. Two of our study participants were teaching staff at the bio-dental materials department, which is an academic department, however, they do have a private clinic. General dentists were excluded in accordance with Nikdel et al., 2018 as dentists in dental centers or private offices who experience an error-in the best of cases, they learn from it, but in most cases, they will try to conceal it from other professionals [7]. This means that a bulk of information on errors will be lost and cannot be properly studied.

An electronic Questionnaire was utilized for data collection due to the wide range of accessibility offered to participants through social media and messaging apps [23]. To promote participants’ reporting, a simple form causes the least inconvenience and ensures reporter confidentiality was adopted [24–26].

As an initial approach, we needed to know whether the study participants recognize the problem of errors and if they believe that it represents an issue that needs to be dealt with. 94% of our participants agreed upon this issue. An interesting finding was reported in this work, as lecturers were significantly more aware than professors in believing that this is an urgent problem that needs to be managed. Further, those of Al-Azhar university were more aware of the issue than private universities participants, we guess that this could be attributed to the lack of resources at Al-Azhar university compared to the private ones, made errors more obvious .

A high prevalence of errors was reported, nearly 8% of participants reported that it represents around 40–60% of cases they see and nearly half of them reported that diagnostic errors represent 20–40%. National Practitioner Data Bank in 2013 reported that diagnosis-related dental allegations were 10% of total claims [27], other data reported that adverse events caused by errors in dental diagnosis constituted about 15.7% of total dental adverse effects, however, there is a difference in methods of data collection as they reviewed the legal claims from 2000 to 2010 [20].

We aimed to first identify areas in the dental field that comprise the most seen diagnostic errors to subsequently focus on different conditions included in these areas upon further investigations. In our study, oral mucosal related diseases represented the top commonly seen diagnostic errors conditions, followed by hard tooth structures related conditions followed by temporomandibular joint and periodontal tissue diseases. A high percentage of participants were oral medicine and periodontology specialists, and this could be considered a source of bias, however, this is expected, as specialists generally tend to report cases in their specialty [3]. Further, this finding is supported by previous studies where delays in referral by clinicians are a huge contributing factor to delayed diagnosis of oral cancer [20, 28, 29]. Oral cancer and periodontal diseases were also on the top of the missed and delayed diagnosed dental conditions reported by Nikdel et al. [7]. Regression analysis showed that Al-Azhar and Governmental universities staff reported that oral mucosal lesions were the most diagnostic errors conditions followed by pulp therapy related conditions, disagreeing with the staff of Private universities who reported the pulp therapy related conditions to be the most common diagnostic errors conditions, more data regarding various aspects related to teaching and assessment methods and the contents of both subjects is needed to explain this observation.

The third part of our survey addressed the contributing factors that might cause dental diagnostic errors, in addition to the known system-related and cognitive-related factors, Nikdel et al. added patient-related factors [7]. In our questionnaire, we didn’t include a direct choice regarding patient-related factors, as we believe that understanding the patient complaint correctly and proper communication with the patient is a skill and cognitive ability of the dentist, further, and in support of this belief, none of our participants pointed out to patient-related factors in other possible causes of diagnostic errors.

Egypt is one of the low-and-middle-income countries [30] where diagnosis poses even greater challenges as the process is more twisted by limited access to care and diagnostic testing resources, insufficient healthcare professionals due to lack of training, outward migration or poor employment situation- plus the illiteracy of some patients that creates barriers against effective communications with the health care providores [13, 14]. Our results further support the interplay between an individual’s cognitive and system-related factors in causing diagnostic errors. Lack of resources, misconduct in medical education strategies, and lack of postgraduate training were the main causes of diagnostic errors in dentistry.

Much attention has been given to the function of healthcare systems as a cause of diagnostic errors, however, little has been done to address the cognitive component of diagnostic errors [21, 31]. It was long believed that undergraduate education in certain dental specialties like pediatric dentistry would prepare general dentists for adequately treating children [32]. The authors-who are dental teaching staff with considerable clinical experience- noticed that diagnostic errors are considerably associated with pitfalls in knowledge, critical thinking, and reasoning among many dental practitioners, thus we put a direct question asking the participants about the role of education in causing errors, nearly 61% of participant believed that medical education strategies are blamed for diagnostic errors. In this regard, Royce et al., 2019 argued that teaching critical thinking skills may have a valuable role in reducing diagnostic errors and improving patient safety [33].

Our study participants reported that a percentage of the errors were committed by themselves as well as by others, more data are needed with a tailored design to know ‟what went wrong” and “why did it happen” to offer those committing an error, the opportunity to frankly share feedback in a blame-free context. As for the clinical impact of the errors, the minor and moderate levels of impact were the most prevalent. Indeed, ratings of error seriousness by dentists might be subjective and mixed judgments about the seriousness of the outcome or diagnosis as well as the error itself. However, all errors should be given adequate attention, as they have the potential to cause mistreatment, reduce a patient’s quality of life, and pose a financial burden to the patient [7].

Considerable variability was noted among responses regarding other problems in the dental field that need to be addressed including improper treatment, the need for patient education, the need for patient screening, medical insurance coverage, etc. These responses represent areas of further investigations and analysis.

Finally, Diagnosis errors are challenging to detect and dissect, and the individual clinician’s decision should not be the only source of data but also the influence of systems, team members, and patients on the diagnostic process should be included. However, valuable information can be gleaned from even limited data sources as long as those who use the data remain mindful of its limitations for a given purpose [34, 35].

## Study limitations:

As we assume that most of the provided errors are seen after they were committed and there is a possibility that some data are lost to monitoring, however, there is an advantage as the errors which have been spotted are the most obvious or harmful and hence, they are the ones which must concern professionals. Another limitation was the higher number of oral medicine specialists compared to others. Moreover, although sampling via social media is an easy and quick method to reach participants, however, many of them just did not participate after the invitation in this regard, we think that direct interviews might be of value in further studies addressing this issue despite the bias that might be encountered. Our study was conducted in an academic setting; thus, generalizability issues might be encountered.

## Conclusion

Dental diagnostic errors need attention from all stakeholders in the healthcare system and dental educational facilities. Based on our findings, further studies are needed in oral medicine, dental restorative, and periodontology specialties to further explain the high frequency of diagnostic errors within and to develop mitigation approaches. Finally, the development of efficient and effective teaching strategies is required to improve clinical competence among dental practitioners.

## Supplementary Information

**Additional file 1. **Questionnaire for dental staff about facing the problem of diagnostic errors in dentistry.

## Data Availability

The datasets generated and/or analyzed during the current study are available from the corresponding author on reasonable request.
