# Supplementary material for: Diagnostic errors in Dentistry, opinions of egyptian dental teaching staff, a cross-sectional study
Source: BMC Oral Health. 2022 Dec 20;22:621. doi: 10.1186/s12903-022-02565-9 (PMC9764576; doi:10.1186/s12903-022-02565-9)
Supplement: Supplementary file 1 — Additional file 1. Questionnaire for dental staff about facing the problem of diagnostic errors in dentistry. [file 12903_2022_2565_MOESM1_ESM.docx]

**Questionnaire for dental staff about facing the problem of diagnostic errors in dentistry**

Email: …………………………………………

**Section Ⅰ:**

1- Academic degree.

Mark only one oval.

- Professor
- Associate professor
- Lecturer
- Assistant lecturer

2- Age:

Mark only one oval.

- 25 - 35
- 36 - 50
- Above 50

3- Sex:

Mark only one oval.

- Male
- Female

4- dental specialty:

Mark only one oval.

- Oral medicine and periodontology
- Oral surgery
- Orthodontics
- Pediatric dentistry
- Oral radiology
- Fixed prosthodontics
- Removable prosthodontics
- Conservative dentistry
- Endodontic
- Other:

………………………………….

5- institution:

Mark only one oval.

- Governmental university.
- Private university
- Azhar university

6- Average number of patients you see per week.

Mark only one oval.

- 10 - 20
- 20 - 30
- more than 30 patients

**Section II**

1- Do you believe that dental diagnostic errors represent an urgent problem that needs to be addressed?

Mark only one oval.

- Yes
- No

2- What is your estimated percentage of diagnostic errors you see in your clinical practice?

Mark only one oval.

- less than 20%
- 20% - 40%
- 40% - 60%
- More than 60%

3- Which of the following areas/ aspects include (comprise) the most dental diagnostic errors condition (Please choose 3 choices)

Check all that apply.

- Oral mucosal lesions
- Hard tooth structure-related conditions
- TMJ-related conditions
- Periodontal conditions
- Pulp therapy-related conditions
- Other:

………………………………….

4- Among the areas you choose, please specify the most seen condition, and answer with 1, 2, or 3.

Mark only one oval per row.

|  | Oral mucosal lesions | Hard tooth structure-related conditions | TMJ-related conditions | Periodontal conditions | Pulp therapy-related conditions |
| --- | --- | --- | --- | --- | --- |
| Represents the top commonly seen conditions |  |  |  |  |  |
| Represent the 2^nd^ commonly  Seen conditions |  |  |  |  |  |
| Represents the 3^rd^ commonly seen conditions |  |  |  |  |  |

**Section III:**

1- Do you believe that medical education methodology is one of the factors that lead to diagnostic errors in dentistry?

Mark only one oval.

- Yes
- No
- Do not know

2- What are the possible causes of the diagnostic errors that you see in your clinical practice (please check on the possible causes).

Check all that apply.

- Lack of resources required for proper diagnosis (for example radiographic machines, equipped dental units, etc.….
- Lack of post-graduate training.
- Misconduct in medical education methods for undergraduate students.
- Other:

………………………………………

3- Among the possible causes you chose, please put them in order where:

Mark only one oval per row.

|  | Lack of resources required for proper diagnosis (for example radiographic machines, equipped dental units, etc.…. | Lack of post-graduate training. | Misconduct in medical education methods for undergraduate students. |
| --- | --- | --- | --- |
| Represents the top cause |  |  |  |
| Represent the 2^nd^ ordered cause |  |  |  |
| Represents the 3^rd^ ordered cause |  |  |  |

4- Who made the errors you see and check the percentage.

Mark only one oval per row.

|  | less than 20% | 20% - 40% | 40% - 60% | More than 60% |
| --- | --- | --- | --- | --- |
| Myself |  |  |  |  |
| Other |  |  |  |  |

5- How serious was the clinical impact of the diagnostic errors?

Mark only one oval.

- No impact
- Minor (patient inconvenience, dissatisfaction)
- Moderate (short-term morbidity, higher level of care, invasive procedure
- Major (death, permanent disability, or near life-threatening event)

6- What are the other problems related to dental clinical practice that you face and need to be addressed by the decision-makers?

………………………………………………………………………………………………………………………………………………………………………………………………………………………………………………………………………………………………………………………
